# Trends in Falls and Medicine Use in Australian Long‐Term Care, 2014–2022: Evidence From 8298 Residents

**DOI:** 10.1111/ajag.70240

**Published:** 2026-09-24

**Authors:** Ying Xu, Johanna I. Westbrook, Isabelle Meulenbroeks, Maria C. Inacio, Gillian E. Caughey, Peter D. Hibbert, Jeffrey Braithwaite, Nasir Wabe

**Affiliations:** ^1^ Australian Institute of Health Innovation, Faculty of Medicine, Health and Human Sciences Macquarie University Sydney New South Wales Australia; ^2^ Registry of Senior Australians (ROSA) South Australian Health & Medical Research Institute Adelaide South Australia Australia; ^3^ IIMPACT in Health, Allied Health and Human Performance University of South Australia Adelaide South Australia Australia

**Keywords:** accidental falls, anti‐anxiety agents, antipsychotic agents, hypnotics and sedatives, long‐term care

## Abstract

**Objective(s):**

To evaluate changes in falls and medication‐related quality indicators (QIs) including high sedative load, antipsychotic use and anti‐anxiety or hypnotic medications in long‐term care facilities (LTCFs), and to examine their alignment with major national reforms and COVID‐19 in New South Wales, Australia.

**Methods:**

A repeated cross‐sectional study was conducted using electronic health data from 27 LTCFs (8298 residents). From Jul 2014 to Jun 2022, quarterly prevalence estimates of four QIs were evaluated adjusting for potential confounders. Joinpoint regression analysis was used to identify QI trend changes. Average quarterly percentage changes for the study period were estimated.

**Results:**

The quarterly prevalence of residents who had any falls (28.5%–33.3%) and injurious falls (14.5%–18.6%) remained stable during the study period. The prevalence of residents who had falls requiring hospitalisation varied (3.8%–9.1%) and average quarterly percentage increase was 1.01% (95% confidence interval (CI) 0.30, 2.08). There were changes in quarterly prevalence of residents experiencing high sedative load (28.1%–32.1%), using antipsychotic(s) (8.9%–19.3%), and anti‐anxiety and hypnotic medication(s) (11.4%–19.0%). Average quarterly percentage decreases were at 0.17% (95% CI 0.01, 0.35) in residents experiencing a high sedative load, 2.14% (95% CI 1.90, 2.41) for antipsychotics use and 1.63% (95% CI 1.45, 1.82) for anti‐anxiety or hypnotic medication use. Regarding trend changes, medication‐related QIs decreased from Q3 or Q4 2018, or Q1 2019, around when the Royal Commission was established. Medication‐related QIs continued to decline when restrictions on risperidone were applied and COVID‐19 started.

**Conclusions:**

Medication‐related QIs decreased during the study period, especially after national reforms.

## Impact Statements

Beyond monitoring fall rates, it is also important to evaluate the implementation of evidence‐based‑falls‐prevention processes and strategies in long‐term care facilities in Australia. Ongoing monitoring of high‐risk medicine prescribing remains essential.

## 1 Introduction

The Royal Commission into Aged Care Quality and Safety was established on 8 October 2018. As the highest national form of public inquiry, their interim and final reports identified significant deficits in care and services provided to older people in residential Long‐Term Care (LTC) Facilities (LTCFs) and a limited capacity to monitor and use information to improve the safety and quality of care [1]. Following findings of the Royal Commission, Australia launched the National Aged Care Mandatory Quality Indicator Program (QI Program), making quality indicators (QIs), such as rates of falls and medication use, publicly visible and comparable. Meanwhile, restrictions on risperidone were applied in the Pharmaceutical Benefits Scheme (PBS). From January 2020, individuals with Alzheimer's disease are only qualified for subsidised treatment of risperidone once in every 12 months for behavioural and psychological symptoms, and the use was restricted to ≤ 12 weeks.

Using existing data routinely collected by 27 not‐for‐profit LTCFs in New South Wales (NSW), Australia, we examined four QIs that are included in national and international QI programs and address the most prevalent sources of harm in LTCFs [2], including falls, high sedative load, use of antipsychotics and use of anti‐anxiety or hypnotic medications. These national and international QI programs were identified through our prior work [3, 4]. We aimed to describe temporal trends and trend changes in these four QIs and the alignment of the above‐mentioned three policy initiatives and COVID‐19 pandemic with identified trend changes.

## 2 Methods

### 2.1 Study Design and Setting

We conducted a repeated cross‐sectional study using routinely collected data from 27 LTCFs managed by a not‐for‐profit aged care provider in NSW, Australia. This provider ranked among the top 25 of 636 LTC providers nationally in the financial year of 2025 based on the number of operational places (beds), and among the small proportion (1.7%) of LTC providers who operate over 1000 places (beds) [5]. In each financial year since 2018, it maintained a capacity of over 1000 beds [5]. Those 25 largest LTC providers accounted for approximately 43% of total Australian Government funding for LTCs across the financial years 2019–2022 [5]. One LTCF was in an inner regional area and 26 were in major cities [6]. The study period was from 1 July 2014 to 30 June 2022. Databases for deidentified residents' profiles (e.g., year of birth, sex and entry and departure dates), incident reports and medication administration were obtained from the provider. Ethics was approved by the Macquarie University Human Research Ethics Committee (Project ID: 12513). The authors assert that all procedures contributing to this work comply with the ethical standards of Australia's National Statement on Ethical Conduct in Human Research (2023) and with the Helsinki Declaration of 1975, as revised in 2008. A waiver of consent was granted by the Macquarie University Human Research Ethics Committee for this type of work.

### 2.2 Study Sample

All residents aged ≥ 65 years who were in the facility during the study period and stayed at least 1 day in a quarter were included in the analyses for that quarter. We excluded 511 residents, median age 85 years (interquartile range (IQR) 79–90), who did not have medication administration data during their stay. Residents with conditions of schizophrenia, paranoid or psychotic states, or Huntington's disease recorded at entry to a LTCF (*n* = 455, 5.5%) were not considered in the analyses of high sedative load and antipsychotic medication use [7].

### 2.3 Quality Indicators

The QIs were estimated for each calendar quarter in the study period. Prevalence of residents with any falls, injurious falls and falls requiring hospitalisation, in each quarter were calculated. Falls were ascertained using the provider's standardised electronic incident report forms, which capture all falls experienced by residents. Falls involving injuries by body regions (e.g., head, facial and limb) were documented and classified as injurious falls. Falls requiring hospitalisation were recorded in the electronic system when residents were considered by the care staff as requiring hospital inspection following a fall and not the actual number of people who attended hospital [8].

Prevalence of medication‐related QIs in each quarter (i.e., high sedative load, use of antipsychotics and use of anti‐anxiety or hypnotic medications) were ascertained using the aged care provider's clinical information system, with documented daily medications administered to residents. Anatomical Therapeutic Chemical classification codes were used to identify relevant medications. Sedative load was calculated by summing the sedative rating of each different sedative medication administered in each quarter, and high sedative load was defined as a sedative score of ≥ 3 [9, 10]. Any administration of antipsychotic medication excluding lithium (N05A, but not N05AN01) and anti‐anxiety or hypnotic medication excluding melatonin (N05B or N05C, but not N05CH01) were identified [10, 11].

### 2.4 Statistical Analysis

Logistic regression was adopted to estimate prevalence of QIs. Risk‐adjusted QIs considered covariates, which included age, sex, resident care type (i.e., a categorical variable of permanent, respite and interim), dementia status and average daily number of medications. Sex and dementia status were recorded at residents' entries to LTCFs. Age was calculated based on a resident's year of birth. Age, resident care type, average daily number of medications were time‐dependant variables, that is, their values could change from quarter to quarter for a resident. Facility and length of stay in each quarter were the other two time‐dependent variables. The average daily number of medications was calculated as the total number of regular and PRN (as‐needed) medications for each resident in a quarter divided by the number of days they stayed in the facility. Dietary supplements were not included, and medications included in the specific medication‐related QIs were not excluded from this count, as this covariate represents overall medication burden. Residents could move between facilities during the study period. When they moved, facility was documented as the one they spent more time in that quarter, and data from this facility were used, and no residents were double counted in a quarter. Facility was used as a cluster variable, so that the estimated standard errors of quarterly prevalence allowed for intra‐facility correlations. Length of stay in each quarter was transformed to a logarithm with base 10 and used as an offset. All QIs were analysed separately for those with and without dementia.

Joinpoint regression analyses were used to identify inflection points, that is., significant changes in trends [12]. Risk‐adjusted QIs were assumed to change at a constant percentage per quarter in each segment, separated by inflection points. Quarterly percentage changes with 95% confidence intervals (CIs) were estimated for all segments, considering standard errors of the performance. Average quarterly percentage changes for the entire study period were estimated as weighted averages of quarterly percentage changes, with weights equal to the length of segments. Acknowledging overlapping effects of progressively implemented policy reforms combined with COVID‐19 lockdowns, Joinpoint regression analyses were adopted over interrupted time series analyses because pre‐specified interruption points were not required.

Stata MP 18 (StataCorp LP, College Station, TX) was used for descriptive analyses and prevalence estimations. Joinpoint regression analyses were conducted in Joinpoint 5.0 (United States National Cancer Institute).

### 2.5 Policy Initiatives and COVID‐19 Pandemic

We examined the alignment of three policy initiatives and the COVID‐19 pandemic with statistically identified trend changes. First, the Royal Commission, held from October 2018, was included as it contained a public hearing process to gather evidence from stakeholders, encouraging and solving complaints and increasing public awareness [1]. LTCFs with resources (e.g., electronic health records) and willingness may proactively initiate improvements [13]. Second, Australia's QI Program, from its expansion in July 2021, was considered because it mandated and publicly reported the proportion of residents experiencing falls and receiving antipsychotics. The transparency of care quality in the QI program allows consumers to choose higher‐quality facilities and incentivises providers to improve performance. Finally, the PBS restrictions on risperidone, introduced in 2020, were included, as limiting long‐term prescribing of this antipsychotic is likely to impact sedative load and antipsychotic use. In addition to policy considerations, we also considered the COVID‐19 pandemic (Q1 2020). Prior Australian and international evidence suggests it influenced rates of falls and prescribing of high‐risk medications [8, 14].

## 3 Results

A total of 8298 residents (24–1081 residents/LTC, median 295, IQR 165–464), with a median age 87 years, IQR 81–91, 64.3% female residents and 46.3% with dementia, were analysed. Table S1 showed characteristics of residents included in the 8‐year observation. In each of the 32 quarters, between 1572 and 2309 residents were analysed.

### 3.1 Averaged Quarterly Percentage Changes From Q3 2014 to Q2 2022

#### 3.1.1 Falls

**FIGURE 1:**
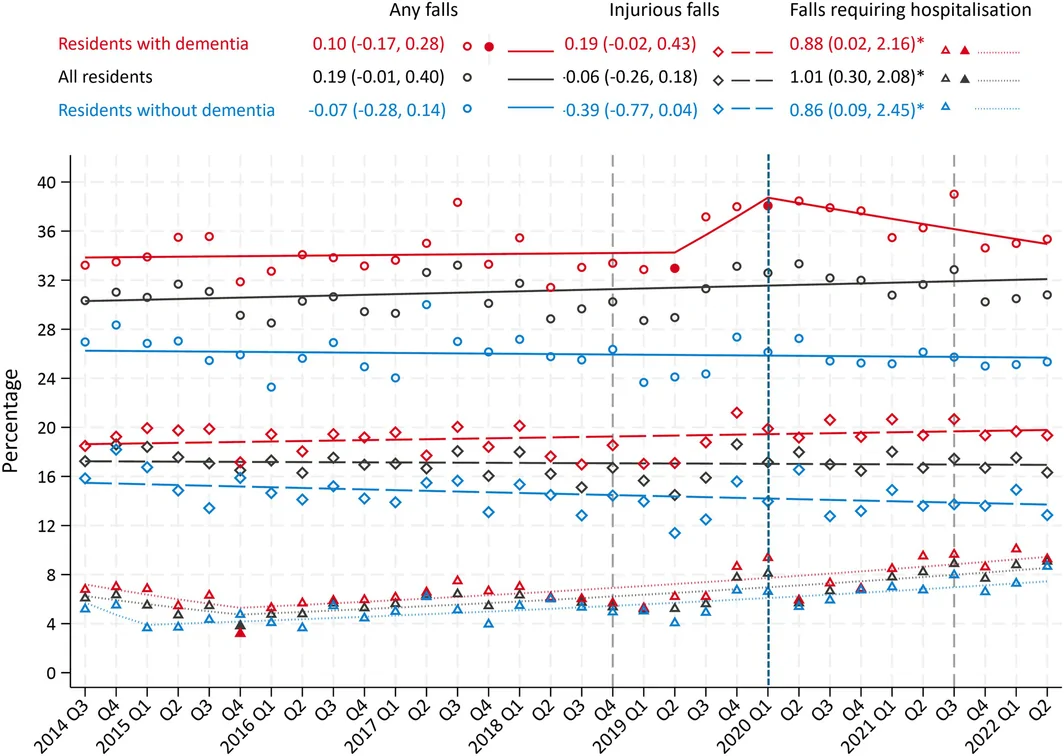
Quarterly measurements of falls. Prevalence of residents who had falls, injurious falls and falls requiring hospitalisation. Lines were fitted using Joinpoint regression analyses. Solid makers indicate inflection points leading segments with significant quarterly percentage changes. Estimated quarterly percentage changes with 95% confidence intervals were reported in Table S2 for each segment. Average quarterly percentage changes with 95% confidence intervals were reported on the top of the graph and in units of %. *significant changes. Models were adjusted for age, sex, resident care type (i.e., permanent, respite and interim care), dementia (when not stratified by dementia status) and averaged daily number of medications in each quarter. Length of stay in each quarter was transformed to a logarithm with base 10 and used as an offset. Facility was used as a cluster variable. Vertical dash lines mark the establishment of the Royal Commission into Aged Care Quality and Safety in October 2018, COVID‐19 in March 2020, and the first expansion of the National Aged Care Mandatory Quality Indicator Program in July 2021.

Between Q3 2014 and Q2 2022, there were no changes in the prevalence of residents who experienced falls and injurious falls (Figure 1). The prevalence of residents who had falls requiring hospitalisation increased 1.01% (95% CI 0.30, 2.08) per quarter. The increases were 0.88% (95% CI 0.02, 2.16) per quarter for residents with dementia and 0.86% (95% CI 0.09, 2.45) for residents without dementia.

#### 3.1.2 Medication‐Related QIs

**FIGURE 2:**
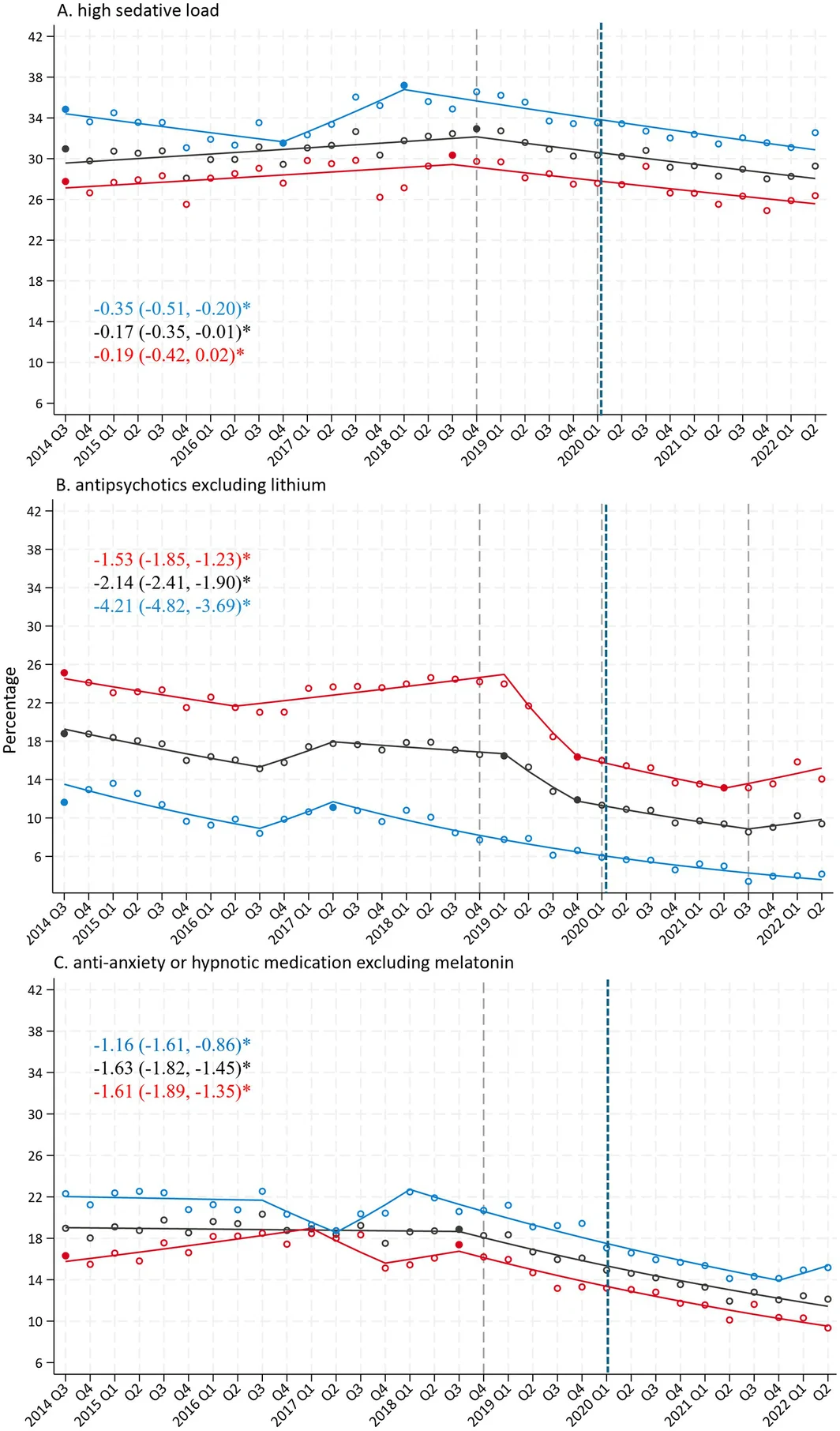
Quarterly measurements of medication‐related quality indicators. Prevalence of residents who had high sedative load (A) and used any antipsychotics (B) and anti‐anxiety or hypnotic medications (C). Excluded residents with conditions of schizophrenia, paranoid or psychotic states, or Huntington's disease recorded at an entry to a residential long‐term care facility for (A) and (B). Lines were fitted using Joinpoint regression analyses. Solid makers indicate inflection points leading segments with significant quarterly percentage changes. Estimated quarterly percentage changes with 95% confidence intervals were reported in Table S2 for each segment. Average quarterly percentage changes with 95% confidence intervals were reported in the graph and in units of %. *significant changes. Models were adjusted for age, sex, resident care type (i.e., permanent, respite and interim care), dementia (when not stratified by dementia status) and averaged daily number of medications in each quarter. Length of stay in each quarter was transformed to a logarithm with base 10 and used as an offset. Facility was used as a cluster variable. The vertical dash lines mark (A) the establishment of the Royal Commission into Aged Care Quality and Safety in October 2018, the Pharmaceutical Benefits Scheme restrictions on risperidone in January 2020, and COVID‐19 in March 2020; (B) the establishment of the Royal Commission into Aged Care Quality and Safety in October 2018, the Pharmaceutical Benefits Scheme restrictions on risperidone in January 2020, COVID‐19 in March 2020, and the first expansion of the National Aged Care Mandatory Quality Indicator Program in July 2021; (C) the establishment of the Royal Commission into Aged Care Quality and Safety in October 2018, and COVID‐19 in March 2020.

Decreases were observed in all three medication‐related QIs (better performance) over the study period for all residents and for subgroups of residents with and without dementia (Figure 2). Decrease in use of antipsychotic(s) was steeper for residents with dementia at 4.21% per quarter (95% CI 3.69, 4.82) than for residents without dementia at 1.53% (95% CI 1.23, 1.85).

### 3.2 Inflection Points and Quarterly Percentage Changes for Segments

#### 3.2.1 Falls

There were trend changes in the prevalence of residents with dementia who had any falls in Q2 2019 and Q1 2020 (Figure 1). In the three quarters between these two inflection points, there was an increase of 4.18% (95% CI 0.88, 5.95) per quarter (Table S2). After Q1 2020, there was a quarterly decrease of 1.13% (95% CI 0.41, 3.28). There were trend changes in Q4 2015 for falls requiring hospitalisation, after which steady increases occurred (Figure 1 and Table S2). The increases were 2.29% (95% CI 1.58, 5.49) per quarter for all residents and 2.26% (95% CI 1.26, 7.52) per quarter for residents with dementia. The identified inflection points did not match with the Royal Commission or the QI Program, but the trend change in Q1 2020 leading to decreases in falls among residents with dementia occurred concurrently with the start of COVID‐19.

#### 3.2.2 Medication‐Related QIs

In Figure 2A and Table S2, for high sedative load, there were trend changes in 2018. These occurred in Q1 2018 for residents without dementia, Q3 2018 for residents with dementia and Q4 2018 for all residents. Following these trend changes, there were decreases in the prevalence of residents with a high sedative load exposure, at 0.97% (95% CI 0.58, 1.66) per quarter for all residents, 0.93% (95% CI 0.49, 1.88) for those with dementia and 1.03% (95% CI 0.78, 1.33) for those without dementia. These trend changes led to significant decreases in the year when the Royal Commission was established (Q4 2018).

Inflection points leading to a decrease in prevalence of residents who used antipsychotics occurred in Q1 2019 for all residents and in Q4 2019 for residents with dementia, after which decreases were at 11.01% (95% CI 0.56, 12.81) per quarter and 3.70% (95% CI −0.77, 11.92), respectively (Figure 2B and Table S2). Decreases in antipsychotics use ended in 2021, in Q2 for residents with dementia and Q3 for all residents. An inflection point in Q2 2017 was identified for residents without dementia, followed by 5 years of decline in antipsychotics use. Trend changes leading segments with significant decreases observed in all residents (Q1 2019) and in residents with dementia (Q4 2019) occurred in the year after the Royal Commission was established (Q4 2018).

Decreases in the use of anti‐anxiety or hypnotic medications for all residents and residents with dementia started from Q3 2018 at 3.21% (95% CI 2.79, 3.79) per quarter and 3.70% (95% CI 1.91, 4.68), respectively. Trend changes leading segments with significant decreases in anti‐anxiety or hypnotic medications in all residents and those with dementia occurred concurrently with the establishment of the Royal Commission (Q4 2018).

## 4 Discussion

In this 8‐year study, the proportions of residents who had a fall and an injurious fall remained stable while the proportion of falls requiring hospitalisation increased. High sedative load, antipsychotic use and anti‐anxiety or hypnotic medications decreased in all residents and residents with dementia from Q3 or Q4 2018, or Q1 or Q4 2019 around or after the year when the Royal Commission was established.

In our analysis, there was no change in falls rate after the introduction of the QI Program, which specifically reported on the proportion of residents experiencing a fall. Australian national data sets have also not detected a consistent decrease in falls in LTCFs after the introduction of the QI Program [15, 16]. The lack of impact on the proportion of falls in LTCFs after the introduction of the QI Program is consistent with some international experiences. In Canada, no changes were found comparing the trend of falls 2011–2014 to that of 2015–2018, after prevalence data of falls in each LTCF became publicly available from 2015 [17].

Despite the lack of improvement in all falls in our analysis post policy introduction, it is well established that falls in LTCFs can be prevented. Gold standard falls prevention interventions in LTCFs, exercise interventions, alongside environmental and medication reviews [18], have demonstrated an up to 55% reduction in the number of fallers a year [19]. Therefore, the lack of change demonstrated in our study and Australian‐wide data [15, 16] could be due to two key factors. First, it is too early to conclusively determine whether the Australia's policy changes have had an impact on the proportion of residents experiencing a fall. Changes in health impact, such as falls, occur after processes, such as falls prevention activities have been changed. This pipeline, from process change to health outcome impact, takes time to establish and may already be underway in Australian LTCFs but is undetected in QI measures as they do not measure falls prevention processes, for example, exercise group attendance, medication review activity. A delayed response to the introduction of a fall's QI has been seen internationally. In the United States, a QI which reported on falls in community dwelling adults (self‐reported and collected via survey) found that after a falls prevention initiative was launched in 2012, the rate of falls increased at first before finally decreasing years later in 2016–2018 [20]. This raises an important second factor, simply reporting the proportion of people who fall per quarter as a QI may not be enough to overcome traditional barriers to implementing evidenced‐based falls prevention strategies in LTCFs, such as availability of allied health staff to deliver the intervention and care staff to support fall prevention activities in routine care. In the future Australia may need additional falls prevention policies, beyond public reporting to support the uptake of falls prevention interventions to drive a reduction in falls.

Evidence on the falls rates during COVID is mixed. Some studies suggested increased proportions of residents who had falls during COVID compared to pre‐COVID, possibly due to staffing shortages and disruptions to usual care. For instance, analysing data from the same provider, increases in monthly rates of falls were found between March to June 2020 (the first lockdown in NSW) compared to the 1‐year pre‐pandemic period [8]. Yet, expanding the observation window to a much longer timeframe revealed no corresponding increase in quarterly fall rates in the current study. Although residents' characteristics were statistically accounted for in both the prior and current work, with an expanded study period, residual confounding is more likely to have occurred and influenced the trend changes in the current work. Conversely, lowered opportunities for falls due to reduced group activities and visitor restrictions may lead to reduced falls rates in the short term and reduced fall‐related hip fracture hospitalization rates have been reported [21].

In this study, the prevalence of people experiencing a fall‐related hospitalisation increased despite the prevalence of residents who had falls remaining constant. This suggests that either the population is becoming frailer and experiencing more serious falls, or that there is an increase in potentially inappropriate transfers to hospital following a fall. There is limited international evidence on potentially inappropriate transfer rates over time. However, a systematic review identified that up to 55% of transfers to hospital from LTCFs are potentially unnecessary and falls are a significant cause of potentially unnecessary transfer [22]. In Australia, the observed increase in hospitalisation post fall could be an unintended consequence of well‐intentioned care staff who were aiming to deliver high quality care to the resident. Further research is required to explore this costly trend in care.

In our analysis, we identified large decreases in antipsychotic use. Such decreases in antipsychotic use were also observed after policy changes internationally. In the United States, the 2012 National Partnership to Improve Dementia Care in Nursing Homes and the 2015 Five Star Quality Ratings coincided with the decreases in antipsychotic prescribing between 2011 and 2017 [23]. In Ontario, Canada, after the Long‐Term Care Homes Act in 2010 and gradually implemented public reporting, there were decreases in antipsychotic use without diagnosis in LTCFs for around 4 years [24]. In the United Kingdom, after the 2006 National Institute for Health and Care Excellence guidance, a steady 8‐year reduction in prevalence of antipsychotic use in older adults with dementia was reported [25]. Our analyses captured antipsychotic use without indications and the decreases in use occurred as early as Q2 2017 among residents without dementia. Relevant to the three QIs we investigated, potentially inappropriate use of psychotropics in Sydney LTCFs and advocates for non‐pharmaceutical interventions have been raised up decades ago [26]. In residents without dementia, fewer behavioural symptoms and better understanding of the benefits of antipsychotic deprescribing resulting in lower risk of relapse after withdrawal [27], may have led to a decrease long before the Royal Commission and a greater overall decline among residents without dementia than those with dementia. These averaged quarterly declines transferred to declines by 73.6% in residents without dementia compared to 34.6% in those with dementia in our 8‐year study period. A recent study using administration data from 428 LTCFs in Australia did not observe such differences [28]. Between January 2018 and December 2022, antipsychotic use decreased by 31.7% and 34.6% in residents without and with a documented dementia, respectively [28]. Yet, unlike the current study, residents with psychoses for whom there is a legitimate indication for antipsychotics were not excluded [28]. The differences in identified inflection points are likely due to variations between practice in aged care providers and facilities, although study period and measurement frequency (quarterly versus monthly) also differ.

Consistent with the national level data [28], decreases in medication‐related QIs remained in these 27 LTCFs towards the end of the study with no further steeper declines. Restrictions on risperidone and COVID‐19 both occurred in Q1 2020. COVID‐19 did not seem to affect anxiolytics and hypnotics use, which is consistent with international evidence [29]. Our findings of decreases in sedative load and anti‐anxiety or hypnotic medications use imply no or limited swapping from antipsychotics including risperidone due to tighter regulatory monitoring and towards anxiolytic, selective serotonin reuptake inhibitors, tiapride, trazodone, carbamazepine, sertraline and analgesics for their sedative impacts [30]. There were no such switches in the United States after two initiatives to reduce antipsychotics use [23]. In agreement with the Australian national level data, although antipsychotic use was higher among residents with dementia, anti‐anxiety or hypnotic medications were lower in these residents [28].

Increases in psychotropic medication use during COVID compared to pre‐COVID have been reported in geriatric units of nursing homes in Spain and among nursing home residents in Canada and the United Kingdom [31, 32, 33, 34]. Yet, unchanged rates of psychotropic medications use were also reported between pre‐ and during COVID periods among residents in nursing homes and assisted living residents in the United States, with increases observed only for the initiation of use among new admissions [35]. The reduction in use observed in this study may reflect Australia's effective response to COVID [36]. In contrast to previous studies that compared outcomes over a shorter pre‐ and post‐pandemic period, our study spans a longer timeframe during which COVID‐19 occurred alongside multiple policy reforms aimed at reducing inappropriate medication use.

A limitation of our study is that our data did not include linked hospital records, such as emergency department presentations or inpatient admissions. Consequently, we could not verify whether falls recorded as ‘requiring hospitalisation’ resulted in an actual hospital presentation or admission. Second, we could not test the validity of health conditions recorded at LTCFs entries, that is, schizophrenia, paranoid or psychotic states or Huntington's disease and do not have information on any changes for individual residents in these conditions over time. However, the quarterly prevalence of 7% to 10% of these conditions were consistent with the 5.9%, 7.1%, 9.2% and 9.8% reported in earlier studies [37]. A limitation of the risk adjustment is that it only includes age, sex, resident care type, dementia status and average daily number of medications and does not capture other factors known to influence falls and medication use, such as functional status, frailty, comorbidity and staffing levels. If residents became frailer with more comorbidities over the study period, stable falls rates might demonstrate improved care, because falls did not increase despite a higher‐risk population. Similarly, reductions in medication use may reflect changes in prescribing needs rather than practice, for instance, when there were greater proportions of residents receiving palliative or end‐of‐life care. Additionally, our definition, data collection and exclusions for QI calculations are close to the national QI program, but we acknowledge variations between different QI programs [3, 10, 38] (Table S3–S6). Because this study focused on medication classes included in current national and international Quality Indicator Programs, other clinically important medication classes, such as antidepressants and opioids, were not examined and require further research. Notably, national data indicate that antidepressant use has increased [28, 39].

Finally, to ensure that new policies drive quality improvement in LTCFs, it is important to evaluate the effectiveness of widespread care quality reforms in Australian LTCFs, to determine whether the investment has translated to better care and health outcomes for residents. However, we cannot conclude with a causal effect of a single policy change on care, as effects overlapped, with for instance any changes after Q3 2021 occurred during the pandemic. Further, other changes in the aged and health care sectors, and actions within these 27 facilities, may have also affected performance. Although the three policy reforms we investigated were the most relevant to the four QIs we examined, these reforms were implemented gradually along with a series of other reforms. For example, 2 years prior to the first expansion of the National Aged Care Mandatory Quality Indicator Program in July 2021, the national QI program was introduced and required LTCF to monitor and report on pressure injuries, unintended weight loss and use of physical restraints. Monitoring on physical restraints may unintentionally result in providers switching to inappropriate chemical restraints (medication use) during those 2 years.

## 5 Conclusion

The lack of reduction in falls, coupled with rising fall‐related hospitalisations, indicates that Australia's recent policy reforms have not improved fall‐related outcomes in LTCFs. Additional policy action may be needed to support evidence‐based falls prevention strategies in LTCFs, creating a multipronged policy approach to improving fall‐related outcomes. In contrast, Australia's new policy environment, which included multiple strategies to improve prescribing of high‐risk medications, has resulted in continued improvements in medication‐related quality indicators without substitution of other high‐risk medications.

## Funding

The authors thank the South Australian Government Department for Innovation and Skills (20172021) who provided the support to establish ROSA, the Australian Government Medical Research Future Fund (2021–2024 PHRDI000009; 2022–2025 GNT 2015823; and 2024–2029 NCRI000109) and ongoing support. The study sponsors had no role in the design, methods, analysis and preparation of the paper.

J. I. W. was supported by National Health and Medical Research Council (NHMRC) Elizabeth Blackburn Leadership Fellowship (1174021, GNT119378, and GNT2026400).

## Ethics Statement

Ethics was approved by the Macquarie University Human Research Ethics Committee (Project ID: 12513). The authors assert that all procedures contributing to this work comply with the ethical standards of Australia's National Statement on Ethical Conduct in Human Research (2023) and with the Helsinki Declaration of 1975, as revised in 2008. A waiver of consent was granted by the Macquarie University Human Research Ethics Committee for this type of work.

## Conflicts of Interest

The authors declare no conflicts of interest.

## Supporting information


**Table S1:** Characteristics of residents included in each quarter of the 8‐year observation.
**Table S2:** Quarterly percentage changes for each segment from the start of the study or after when there was an inflection point.
**Table S3:** Details on residents who experienced falls in the current study and in comparisons to other quality improvement programs when applicable.
**Table S4:** Details on residents who had a high sedative load in the current study and in comparisons to other quality improvement programs when applicable.
**Table S5:** Details on residents who used antipsychotics in the current study and in comparisons to other quality improvement programs when applicable.
**Table S6:** Details on residents who used anti‐anxiety or hypnotic in the current study and in comparisons to other quality improvement programs when applicable.

## Data Availability

Data are not publicly available but may be available from the authors upon reasonable request and with permission of the aged care provider.
