# Supplementary material for: Trends in Falls and Medicine Use in Australian Long‐Term Care, 2014–2022: Evidence From 8298 Residents
Source: Australas J Ageing. 2026 Sep 24;45(3):e70240. doi: 10.1111/ajag.70240 (PMC13612836; doi:10.1111/ajag.70240)
Supplement: Supplementary file 1 [file AJAG-45-0-s001.docx]

# Supplementary

This supplementary provides additional information for the article: Trends in Falls and Medicine Use in Australian Long-Term Care, 2014–2022: Evidence from 8298 Residents

Contents

[Supplementary	1](#_Toc212792295)

[Table S1 Characteristics of residents included in each quarter of the 8-year observation	2](#_Toc212792296)

[Table S2 Quarterly percentage changes for each segment from the start of the study or after when there was an inflection point	4](#_Toc212792297)

[Table S3 Details on residents who experienced falls in the current study and in comparisons to other quality improvement programs when applicable	5](#_Toc212792298)

[Table S4 Details on residents who had a high sedative load in the current study and in comparisons to other quality improvement programs when applicable	7](#_Toc212792299)

[Table S5 Details on residents who used antipsychotics in the current study and in comparisons to other quality improvement programs when applicable	8](#_Toc212792300)

[Table S6 Details on residents who used anti-anxiety or hypnotic in the current study and in comparisons to other quality improvement programs when applicable	10](#_Toc212792301)

# Table S1 Characteristics of residents included in each quarter of the 8-year observation

| Review period | Number of residents analysed | | | Age (in years), median (Q1, Q3) | | Percentage of female residents | | Averaged daily number of medicines, median (Q1, Q3) | | Percentage of permanent residents | | Length of stay (in days) median (Q1, Q3) | |
| --- | --- | --- | --- | --- | --- | --- | --- | --- | --- | --- | --- | --- | --- |
|  | 1^a^ | 2^a^ | 2/1^a^, % | 1^a^ | 2^a^ | 1^a^ | 2^a^ | 1^a^ | 2^a^ | 1^a^ | 2^a^ | 1^a^ | 2^a^ |
| 2014 quarter 3 | 1869 | 1734 | 92.8 | 92 (86, 92) | 88 (84, 92) | 73.5 | 73.2 | 7 (5, 9) | 7 (5, 9) | 90.8 | 90.4 | 92 (86, 92) | 92 (85, 92) |
| 2014 quarter 4 | 1812 | 1682 | 92.8 | 92 (87, 92) | 88 (83, 92) | 72.9 | 72.5 | 7 (5, 9) | 7 (5, 9) | 91.1 | 90.7 | 92 (87, 92) | 92 (87, 92) |
| 2015 quarter 1 | 1763 | 1635 | 92.7 | 90 (88, 90) | 89 (84, 93) | 73.3 | 73.1 | 7 (5, 9) | 7 (5, 9) | 92.8 | 92.5 | 90 (88, 90) | 90 (88, 90) |
| 2015 quarter 2 | 1748 | 1627 | 93.1 | 91 (87, 91) | 89 (84, 93) | 73.3 | 73.0 | 7 (5, 9) | 7 (5, 9) | 92.6 | 92.1 | 91 (87, 91) | 91 (87, 91) |
| 2015 quarter 3 | 1773 | 1654 | 93.3 | 92 (84, 92) | 89 (84, 93) | 72.9 | 72.5 | 7 (5, 9) | 7 (5, 9) | 90.0 | 89.5 | 92 (84, 92) | 92 (82, 92) |
| 2015 quarter 4 | 1743 | 1628 | 93.4 | 91 (86, 91) | 88 (84, 92) | 72.4 | 72.2 | 7 (5, 9) | 7 (5, 9) | 90.4 | 89.9 | 91 (86, 91) | 91 (86, 91) |
| 2016 quarter 1 | 1744 | 1626 | 93.2 | 91 (87, 91) | 89 (84, 93) | 72.1 | 71.8 | 7 (5, 9) | 7 (5, 9) | 90.5 | 90.2 | 91 (87, 91) | 91 (86, 91) |
| 2016 quarter 2 | 1741 | 1624 | 93.3 | 91 (86, 91) | 89 (84, 93) | 71.2 | 70.9 | 7 (5, 9) | 7 (5, 9) | 89.8 | 89.4 | 91 (86, 91) | 91 (85, 91) |
| 2016 quarter 3 | 1744 | 1626 | 93.2 | 92 (85, 92) | 89 (84, 93) | 70.1 | 69.7 | 7 (4, 9) | 7 (4, 9) | 90.4 | 89.9 | 92 (85, 92) | 92 (84, 92) |
| 2016 quarter 4 | 1747 | 1622 | 92.8 | 92 (84, 92) | 88 (83, 92) | 69.9 | 69.5 | 7 (5, 9) | 7 (5, 9) | 90.2 | 90.0 | 92 (84, 92) | 92 (83, 92) |
| 2017 quarter 1 | 1718 | 1594 | 92.8 | 90 (86, 90) | 89 (84, 93) | 69.4 | 69.0 | 7 (5, 9) | 7 (4, 9) | 91.0 | 90.4 | 90 (86, 90) | 90 (85, 90) |
| 2017 quarter 2 | 1702 | 1572 | 92.4 | 91 (87, 91) | 89 (84, 93) | 69.7 | 69.5 | 7 (5, 10) | 7 (5, 10) | 92.9 | 92.4 | 91 (87, 91) | 91 (86, 91) |
| 2017 quarter 3 | 1832 | 1698 | 92.7 | 92 (66, 92) | 89 (84, 93) | 69.1 | 68.6 | 7 (5, 10) | 7 (5, 10) | 90.6 | 90.1 | 92 (66, 92) | 92 (63, 92) |
| 2017 quarter 4 | 1808 | 1673 | 92.5 | 92 (85, 92) | 88 (83, 93) | 69.7 | 69.3 | 7 (5, 10) | 7 (5, 10) | 90.8 | 90.3 | 92 (85, 92) | 92 (83, 92) |
| 2018 quarter 1 | 1804 | 1661 | 92.1 | 90 (85, 90) | 89 (84, 93) | 70.3 | 69.9 | 7 (5, 10) | 7 (5, 10) | 91.8 | 91.5 | 90 (85, 90) | 90 (85, 90) |
| 2018 quarter 2 | 1839 | 1694 | 92.1 | 89 (69, 90) | 89 (83, 93) | 70.4 | 70.1 | 7 (4, 9) | 7 (4, 9) | 92.4 | 91.9 | 89 (69, 90) | 89 (67, 90) |
| 2018 quarter 3 | 2167 | 2003 | 92.4 | 92 (69, 92) | 89 (83, 93) | 69.9 | 69.8 | 7 (5, 9) | 7 (5, 9) | 90.3 | 89.9 | 92 (69, 92) | 92 (66, 92) |
| 2018 quarter 4 | 2169 | 2001 | 92.3 | 92 (85, 92) | 88 (83, 93) | 69.6 | 69.4 | 7 (5, 9) | 7 (5, 9) | 92.5 | 92.2 | 92 (85, 92) | 92 (85, 92) |
| 2019 quarter 1 | 2180 | 2010 | 92.2 | 90 (86, 90) | 89 (83, 93) | 68.8 | 68.5 | 7 (5, 9) | 7 (5, 9) | 91.7 | 91.2 | 90 (86, 90) | 90 (85, 90) |
| 2019 quarter 2 | 2179 | 2011 | 92.3 | 91 (86, 91) | 89 (83, 93) | 69.1 | 69.0 | 7 (5, 9) | 7 (5, 9) | 91.9 | 91.5 | 91 (86, 91) | 91 (86, 91) |
| 2019 quarter 3 | 2187 | 2019 | 92.3 | 92 (86, 92) | 89 (83, 93) | 69.0 | 68.8 | 7 (4, 9) | 7 (4, 9) | 90.7 | 90.1 | 92 (86, 92) | 92 (85, 92) |
| 2019 quarter 4 | 2203 | 2030 | 92.1 | 92 (87, 92) | 88 (82, 93) | 69.9 | 69.6 | 6 (4, 9) | 6 (4, 9) | 89.9 | 89.7 | 92 (87, 92) | 92 (86, 92) |
| 2020 quarter 1 | 2217 | 2043 | 92.2 | 91 (89, 91) | 89 (83, 94) | 69.6 | 69.3 | 7 (4, 9) | 7 (4, 9) | 91.0 | 90.8 | 91 (89, 91) | 91 (88, 91) |
| 2020 quarter 2 | 2103 | 1932 | 91.9 | 91 (91, 91) | 89 (83, 93) | 70.2 | 69.8 | 7 (5, 9) | 7 (5, 9) | 94.4 | 94.3 | 91 (91, 91) | 91 (91, 91) |
| 2020 quarter 3 | 2112 | 1905 | 90.2 | 86 (84, 87) | 89 (83, 93) | 69.6 | 69.8 | 6 (4, 9) | 6 (4, 9) | 93.6 | 93.5 | 86 (84, 87) | 86 (84, 88) |
| 2020 quarter 4 | 2073 | 1874 | 90.4 | 92 (90, 92) | 88 (82, 93) | 69.5 | 69.9 | 7 (5, 9) | 7 (4, 9) | 93.3 | 92.8 | 92 (90, 92) | 92 (89, 92) |
| 2021 quarter 1 | 2140 | 1942 | 90.7 | 90 (86, 90) | 89 (83, 93) | 69.0 | 69.3 | 7 (4, 9) | 7 (4, 9) | 91.5 | 90.9 | 90 (86, 90) | 90 (84, 90) |
| 2021 quarter 2 | 2224 | 2024 | 91.0 | 91 (85, 91) | 88 (82, 93) | 68.7 | 69.0 | 7 (4, 9) | 7 (4, 9) | 90.0 | 89.3 | 91 (85, 91) | 91 (83, 91) |
| 2021 quarter 3 | 2206 | 2008 | 91.0 | 92 (90, 92) | 88 (82, 93) | 68.6 | 68.7 | 7 (5, 9) | 7 (5, 9) | 90.2 | 89.6 | 92 (90, 92) | 92 (89, 92) |
| 2021 quarter 4 | 2253 | 2042 | 90.6 | 92 (86, 92) | 88 (82, 93) | 68.4 | 68.5 | 7 (5, 9) | 7 (5, 9) | 89.7 | 89.3 | 92 (86, 92) | 92 (86, 92) |
| 2022 quarter 1 | 2254 | 2048 | 90.9 | 90 (88, 90) | 88 (82, 93) | 67.0 | 67.2 | 7 (5, 9) | 7 (5, 9) | 89.9 | 89.6 | 90 (88, 90) | 90 (88, 90) |
| 2022 quarter 2 | 2309 | 2105 | 91.2 | 91 (84, 91) | 88 (82, 93) | 67.7 | 67.8 | 7 (5, 9) | 7 (5, 9) | 90.6 | 90.0 | 91 (84, 91) | 91 (83, 91) |

Q1 denotes lower interquartile limit, and Q3 upper interquartile limit.

^a^Residents needed to have stayed for at least one day in a quarter for that quarter to be included in the analyses, which applied to measurements of falls and anti-anxiety or hypnotic medicine use (marked as study sample 1). Residents with conditions of schizophrenia, paranoid or psychotic states, or Huntington’s disease recorded at an entry to a residential aged care were excluded for the evaluations of high sedative load and antipsychotic medicine use (marked as study sample 2).

# Table S2 Quarterly percentage changes for each segment from the start of the study or after when there was an inflection point

| QI, study sample^1^ | | Quarterly percentage changes with 95% confidence intervals for segments | | | | | |
| --- | --- | --- | --- | --- | --- | --- | --- |
| Falls | 2 | Q3 2014 to Q2 2019 | Q2 2019 to Q1 2020 | Q1 2020 to Q2 2022 |  |  |  |
|  |  | 0.06 (-0.86, 0.34) | 4.18 (0.88, 5.95)* | -1.13 (-3.28, -0.41)* |  |  |  |
| Falls requiring hospitalisation | 1 | Q3 2014 to Q4 2015 | Q4 2015 to Q2 2022 |  |  |  |  |
|  |  | -5.41 (-19.82, 1.48) | 2.29 (1.58, 5.49)* |  |  |  |  |
|  | 2 | Q3 2014 to Q4 2015 | Q4 2015 to Q2 2022 |  |  |  |  |
|  |  | -5.99 (-23.43, 1.70) | 2.26 (1.26, 7.52)* |  |  |  |  |
|  | 3 | Q3 2014 to Q1 2015 | Q1 2015 to Q2 2022 |  |  |  |  |
|  |  | -17.40 (-27.97, 2.50) | 2.25 (-6.18, 6.88) |  |  |  |  |
| High sedative load | 1 | Q3 2014 to Q4 2018 | Q4 2018 to Q2 2022 |  |  |  |  |
|  |  | 0.49 (0.21, 0.91)* | -0.97 (-1.66, -0.58)* |  |  |  |  |
|  | 2 | Q3 2014 to Q3 2018 | Q3 2018 to Q2 2022 |  |  |  |  |
|  |  | 0.51 (0.14, 1.24)* | -0.93 (-1.88, -0.49)* |  |  |  |  |
|  | 3 | Q3 2014 to Q4 2016 | Q4 2016 to Q1 2018 | Q1 2018 to Q2 2022 |  |  |  |
|  |  | -0.92 (-2.34, -0.30)* | 3.05 (1.83, 5.72)* | -1.03 (-1.33, -0.78)* |  |  |  |
| Antipsychotics excluding lithium | 1 | Q3 2014 to Q3 2016 | Q3 2016 to Q2 2017 | Q2 2017 to Q1 2019 | Q1 2019 to Q4 2019 | Q4 2019 to Q3 2021 | Q3 2021 to Q2 2022 |
|  |  | -2.83 (-4.80, -1.11)* | 5.41 (-3.70, 7.29) | -1.02 (-3.44, 4.74) | -11.01 (-12.81, -0.56)* | -3.97 (-9.36, -1.77)* | 3.61 (-0.96, 9.27) |
|  | 2 | Q3 2014 to Q2 2016 | Q2 2016 to Q1 2019 | Q1 2019 to Q4 2019 | Q4 2019 to Q2 2021 | Q2 2021 to Q2 2022 |  |
|  |  | -1.76 (-5.60, -0.12)* | 1.30 (-0.52, 3.84) | -13.04 (-15.33, 1.59) | -3.70 (-11.92, -0.77)* | 3.80 (0.03, 11.00)* |  |
|  | 3 | Q3 2014 to Q3 2016 | Q3 2016 to Q2 2017 | Q2 2017 to Q2 2022 |  |  |  |
|  |  | -5.07 (-12.83, -2.06)* | 9.46 (-4.62, 13.54) | -5.76 (-6.80, -5.00)* |  |  |  |
| Anti-anxiety or hypnotic medicine excluding melatonin | 1 | Q3 2014 to Q3 2018 | Q3 2018 to Q2 2022 |  |  |  |  |
|  |  | -0.12 (-0.47, 0.29) | -3.21 (-3.79, -2.79)* |  |  |  |  |
|  | 2 | Q3 2014 to Q1 2017 | Q1 2017 to Q4 2017 | Q4 2017 to Q3 2018 | Q3 2018 to Q2 2022 |  |  |
|  |  | 1.87 (0.41, 3.26)* | -6.29 (-8.53, 2.65) | 2.40 (-5.86, 4.76) | -3.70 (-4.68, -1.91)* |  |  |
|  | 3 | Q3 2014 to Q3 2016 | Q3 2016 to Q2 2017 | Q2 2017 to Q1 2018 | Q1 2018 to Q4 2021 | Q4 2021 to Q2 2022 |  |
|  |  | -0.20 (-1.51, 2.13) | -5.03 (-7.04, 2.39) | 6.95 (-4.19, 9.10) | -3.20 (-5.20, 5.01) | 4.97 (-2.89, 9.69) |  |

For the study sample, 1 denotes all residents, 2 residents with dementia, and 3 residents without dementia. QI denotes quality indicators.

# Table S3 Details on residents who experienced falls in the current study and in comparisons to other quality improvement programs when applicable

| **Current study or quality improvement programs** | **Definition** | **Data collection** | **Exclusion** | **Risk adjustment** |
| --- | --- | --- | --- | --- |
| Current study | Any: any staff and/or residents reported falls. Injurious: those falls where injured body regions (e.g., head, facial, limb) were documented. Falls requiring hospitalisation were recorded in the electronic system when residents were considered by the care staff as requiring hospital inspection following a fall. | A single review of the standardised electronic incident report forms for each care recipient for the entire quarter. | Residents who were absent from the service for the entire quarter. | Age, sex, resident care type, dementia, and averaged daily number of medicines. Length of stay in each quarter used as an offset. Facility used as a cluster variable. |
| Australian National Aged Care Mandatory Quality Indicator Program | Care recipients who experienced a fall (one or more) at the service during the quarter. | A single review of the care records of each care recipient for the entire quarter. | Care recipients who were absent from the service for the entire quarter. | Differences in numbers of care recipients assessed by each service are considered by including an offset in the model. |
| Registry of Senior Australians Residential Aged Care Outcome Monitoring System | One or more falls requiring medical attention. Number of residents with an emergency department presentation, hospitalisation, death, or injury cause for fall. | Thirty days prior to assessment. | Inpatient falls (onset in hospital). | Age, sex, number of health conditions, dementia, level of mobility. |
| Canada Alberta Health Continuing Care Long Term Care Indicators | Falls in the last 30 days in long term care. | Thirty days prior to quarterly target clinical assessment. | None. | Not totally dependent in transferring, locomotion problem, Personal Severity Index subset 2 (non-diagnoses) (any wandering, unsteady gait/cognitive impairment), age <65 years. |
| Canada Health Quality Ontario Long Term Care Home Performance Quality Indicators | Long-term care home residents who fell in the last 30 days. | Thirty days prior to quarterly target clinical assessment. | None. | Same as the cell above. Additionally, direct standardization: Case Mix Index, and the relative resource use compared to the overall average resource use for all Ontario Long Term Care home residents. |

# Table S4 Details on residents who had a high sedative load in the current study and in comparisons to other quality improvement programs when applicable

| **Current study or quality improvement programs** | **Definition** | **Data collection** | **Exclusion** | **Risk adjustment** |
| --- | --- | --- | --- | --- |
| Current study | Summing the sedative rating (reference 10) of each different sedative medicine administered at least once within each quarter. | Daily medicine administration for each resident for the entire quarter. | Residents with conditions of schizophrenia, paranoid or psychotic states, or Huntington’s disease recorded at entries to an LTC. | Age, sex, resident care type, dementia, and averaged daily number of medicines. Length of stay in each quarter used as an offset. Facility used as a cluster variable. |
| Registry of Senior Australians Residential Aged Care Outcome Monitoring System | Summing the sedative rating of each different medicine dispensed within a 91-day period. | 91-day. | Residents who had reported health conditions of schizophrenia or Huntington’s disease, receiving cancer treatment or are in palliative care. | Age, sex, number of health conditions. Stratified by dementia status. |

LTC denotes residential long-term care facility.

# Table S5 Details on residents who used antipsychotics in the current study and in comparisons to other quality improvement programs when applicable

| **Current study or quality improvement programs** | **Definition** | **Data collection** | **Exclusion** | **Risk adjustment** |
| --- | --- | --- | --- | --- |
| Current study | ATC codes N05A including and excluding N05AN01 that had been administered for at least once within each quarter. | Daily medicine administration for each resident for the entire quarter. | Residents with conditions of schizophrenia, paranoid or psychotic states, or Huntington’s disease recorded at entries to an LTC. | Age, sex, resident care type, dementia, and averaged daily number of medicines. Length of stay in each quarter used as an offset. Facility used as a cluster variable. |
| Australian National Aged Care Mandatory Quality Indicator Program | Care recipients who received an antipsychotic medicine. Care recipients who received an antipsychotic medicine for a diagnosed condition of psychosis. | A seven-day medicine chart or administration record review for each care recipient every quarter. | Care recipients who were admitted to hospital for the entire seven-day assessment period. | Differences in numbers of care recipients assessed by each service are considered by including an offset in the model. |
| Canada Health Quality Ontario Long Term Care Home Performance Quality Indicators | Long-term care home residents without psychosis on antipsychotics. | Seven-day. | Residents who are end-stage disease (<6 mo to live) or receiving hospice care. Residents who have a diagnosis of schizophrenia or Huntington's chorea. Experienced hallucinations or delusions in seven days prior to assessment. | Not totally dependent in transferring, locomotion problem, Personal Severity Index subset 2 (any wandering, unsteady gait/cognitive impairment), age <65 years. Direct standardization: Case Mix Index, and the relative resource use compared to the overall average resource use for all Ontario Long Term Care home residents. |
| Canadian Institute for Health Information Long-Term Care Indicators | Long-term care home residents without psychosis on antipsychotics. | Seven-day. | Same as the cell above. | Motor agitation, moderate/impaired decision-making problem, long-term memory problem, cognitive performance scale, Alzheimer's disease/other dementia, age <65 years.  Facility-Level Stratification: Case Mix Index.  Direct Standardization, Indirect Standardization.  Standard population: 3,000 facilities in 6 states and 92 residential care facilities and continuing care hospitals in Ontario and Nova Scotia. |
| Icelandic Minimum Data Set Quality Indicators | Antipsychotic use in the absence of psychotic and related condition. | 160-day. | Residents with schizophrenia and hallucinations. | Yes, but no details. |

ATC codes denote Anatomical Therapeutic Chemical codes, LTC residential long-term care facility.

# Table S6 Details on residents who used anti-anxiety or hypnotic in the current study and in comparisons to other quality improvement programs when applicable

| **Current study or quality improvement programs** | **Definition** | **Data collection** | **Exclusion** | **Risk adjustment** |
| --- | --- | --- | --- | --- |
| Current study | ATC codes N05B or N05C including and excluding N05CH01 that had been administered for at least once within each quarter. | Daily medicine administration for each resident for the entire quarter. | Residents with conditions of schizophrenia, paranoid or psychotic states, or Huntington’s disease recorded at entries to an LTC. | Age, sex, resident care type, dementia, and averaged daily number of medicines. Length of stay in each quarter used as an offset. Facility used as a cluster variable. |
| United States Centres for Medicare and Medicaid Services Minimum Data Set 3.0 Resident Assessment Instrument Quality Measures | Antianxiety or hypnotic medicine use during the target period. | 90-day. | Any of the following related conditions are present on the target assessment: schizophrenia, Tourette’s syndrome, or Huntington’s disease. | No. |

ATC codes denote Anatomical Therapeutic Chemical codes, LTC residential long-term care facility.
